# Molecular characterization of multidrug-resistant ESKAPEE pathogens from clinical samples in Chonburi, Thailand (2017–2018)

**DOI:** 10.1186/s12879-022-07678-8

**Published:** 2022-08-17

**Authors:** Sirigade Ruekit, Apichai Srijan, Oralak Serichantalergs, Katie R. Margulieux, Patrick Mc Gann, Emma G. Mills, William C. Stribling, Theerasak Pimsawat, Rosarin Kormanee, Suthisak Nakornchai, Chaiwat Sakdinava, Prawet Sukhchat, Mariusz Wojnarski, Samandra T. Demons, John M. Crawford, Paphavee Lertsethtakarn, Brett E. Swierczewski

**Affiliations:** 1 Bacterial and Parasitic Diseases Department, Armed Forces Research Institute of Medical Sciences (AFRIMS), Bangkok, 10400 Thailand; 2 Wound Infections Department, Bacterial Diseases Branch, Walter Reed Army Institute of Research, Silver Spring, MD USA; 3 Multidrug-Resistant Organism Repository and Surveillance Network (MRSN), Walter Reed Army Institute of Research, Silver Spring, MD USA; 4 Queen Sirikit Naval Hospital, Chonburi, Thailand; 5 US Army Medical Research Institute of Chemical Defense, Gunpowder, MD USA; 6 Bacterial Diseases Branch, Walter Reed Army Institute of Research, Silver Spring, MD USA

**Keywords:** MDR, ESKAPEE, CRE, ESBL, *mcr*, Thailand

## Abstract

**Background:**

ESKAPEE pathogens *Enterococcus faecium*, *Staphylococcus aureus*, *Klebsiella pneumoniae*, *Acinetobacter baumannii*, *Pseudomonas aeruginosa*, *Enterobacter* spp. and *Escherichia coli* are multi-drug resistant (MDR) bacteria that present increasing treatment challenges for healthcare institutions and public health worldwide.

**Methods:**

431 MDR ESKAPEE pathogens were collected from Queen Sirikit Naval Hospital, Chonburi, Thailand between 2017 and 2018. Species identification and antimicrobial resistance (AMR) phenotype were determined following CLSI and EUCAST guidelines on the BD Phoenix System. Molecular identification of antibiotic resistant genes was performed by polymerase chain reaction (PCR), real-time PCR assays, and whole genome sequencing (WGS).

**Results:**

Of the 431 MDR isolates collected, 1.2% were *E. faecium*, 5.8% were *S. aureus*, 23.7% were *K. pneumoniae*, 22.5% were *A. baumannii*, 4.6% were *P. aeruginosa*, 0.9% were *Enterobacter* spp*.*, and 41.3% were *E. coli*. Of the 401 Gram-negative MDR isolates, 51% were carbapenem resistant, 45% were ESBL producers only, 2% were colistin resistance and ESBLs producers (2%), and 2% were non-ESBLs producers. The most prevalent carbapenemase genes were *bla*_OXA-23_ (23%), which was only identified in *A. baumannii*, followed by *bla*_NDM_ (17%), and *bla*_OXA-48-like_ (13%). Beta-lactamase genes detected included *bla*_TEM,_ *bla*_SHV_, *bla*_OXA_, *bla*_CTX-M_, *bla*_DHA_, *bla*_CMY_, *bla*_PER_ and *bla*_VEB_. Seven *E. coli* and *K. pneumoniae* isolates showed resistance to colistin and carried *mcr-1* or *mcr-3*, with 2 *E. coli* strains carrying both genes. Among 30 Gram-positive MDR ESKAPEE, all VRE isolates carried the *vanA* gene (100%) and 84% *S. aureus* isolates carried the *mecA* gene.

**Conclusions:**

This report highlights the prevalence of AMR among clinical ESKAPEE pathogens in eastern Thailand. *E. coli* was the most common MDR pathogen collected, followed by *K. pneumoniae*, and *A. baumannii*. Carbapenem-resistant *Enterobacteriaceae* (CRE) and extended spectrum beta-lactamases (ESBLs) producers were the most common resistance profiles. The co-occurrence of *mcr-1* and *mcr-3* in 2 *E. coli* strains, which did not affect the level of colistin resistance, is also reported. The participation of global stakeholders and surveillance of MDR remain essential for the control and management of MDR ESKAPEE pathogens.

**Supplementary Information:**

The online version contains supplementary material available at 10.1186/s12879-022-07678-8.

## Background

ESKAPEE pathogens (*Enterococcus faecium*, *Staphylococcus aureus*, *Klebsiella pneumoniae*, *Acinetobacter baumannii*, *Pseudomonas aeruginosa*, *Enterobacter* spp. and *Escherichia coli*) comprise of high to critical drug resistant pathogens that are the leading cause of hospital acquired infections throughout the world [1]. The Southeast Asian region has a history of a high prevalence of multidrug-resistant (MDR) Gram-negative bacteria that produce extended spectrum beta-lactamases (ESBLs), especially *Escherichia coli, Klebsiella pneumoniae* and *Acinetobacter baumannii* [2, 3]. Additionally, carbapenem-resistant *Enterobacteriaceae* (CRE) have recently emerged and spread widely throughout the region [4]. Gram-positive *Enterococcus* spp. and *Staphylococcus aureus* have also exhibited increasing resistance to last-line antibiotic treatments with the spread of vancomycin-resistant Enterococci (VRE) and methicillin-resistant *S. aureus* (MRSA) [5]. The epidemiology and molecular characteristics of these pathogens with highly resistant phenotypes have been reported in Southeast Asian countries [6], highlighting the urgency of the problem as infections with MDR bacteria are associated with a higher mortality, longer hospital stays, and increased healthcare costs compared to infections with antibiotic susceptible bacteria [7].

In Thailand, MDR bacterial prevalence has been increasing over the past two decades [8, 9]. The detection of carbapenem-resistant *A. baumannii* has increased significantly since 2000, with a current prevalent estimate of up to 80% of carbapenem-resistant *A. baumannii* in several Thai hospitals [10, 11]. Reports from the National Antimicrobial Resistance Surveillance Center, Thailand (NARST) revealed that the percentage of ESBL-producing *E. coli* and *K. pneumoniae* increased from 20 to 40% during 2000–2010 but has remained steady at 48% to 50% during 2011–2020 [9]. The percentage of CRE-producing *E. coli* and *K. pneumoniae* remained relatively low (0.5–1%) from 2000 to 2012 but a steady increase of these pathogens has been observed since 2013 up to 2.8% for *E. coli* and 10.5% for *K. pneumoniae* [9]. The plasmid-mediated colistin resistance gene, *mcr-1*, was first reported in 2015 from a clinical *E. coli* isolate in China [12]. Variants of *mcr* gene have since been reported in several Gram-negative bacterial isolates worldwide, and was first identified in Thailand from a clinical *K. pneumoniae* isolate [13–15]. The global dissemination of *mcr-1* signals a new era of pandrug-resistant bacterial pathogens. A study by Tishyadhigama *et al.* in 2009 reported the prevalence of MRSA remained stable at 26% from 2000 to 2005 [16]. However, a higher estimate of MRSA (65%) has been shown among intensive care unit patients [16, 17]. The percentage of MRSA has decreased from 28% in 2006 to 6.5% in 2020 [9]. The reports from NARST also revealed a high prevalence of vancomycin resistance among *E. faecium,* which has been on the rise since 2010 [9]. Recent studies have also shown a steady increase in the number of vancomycin-intermediate *S. aureus* (VISA) [18].

An annual estimate of 38,000 deaths from a total of 88,000 MDR infections in Thailand cost approximately $1.4 billion annually [19]. Thailand implemented the National Strategic Plan on Antimicrobial Resistance 2017–2021 to combat the rise of AMR through a One Health Approach, which encompasses cross-sectional surveillance and education outreach of antibiotic use in humans, agricultural settings, and the environment. Surveillance and characterization of AMR pathogens, especially ESKAPEE pathogens, plays an essential role in managing the rise of AMR. This study reports the identification and molecular characterization of AMR genes in ESKAPEE pathogens isolated from samples collected from 2017 to 2018 from Queen Sirikit Naval Hospital located in Chonburi province, Thailand.

## Methods

### Bacterial strains and media

A surveillance study of ESKAPEE pathogens was conducted at Queen Sirikit Naval Hospital (QSH) between 2017 and 2018 to identify MDR bacterial strains from routinely collected clinical samples (e.g., pus, urine, rectal swabs, sputum, and blood) (Additional file 2: Table S2) in both inpatient and outpatient setting by VITEK2. MDR was defined as an acquired non-susceptibility to at least one agent in three or more antimicrobial categories (e.g. carbapenems, beta-lactams, and aminoglycosides). ESKAPEE isolates showing MDR characteristics were identified through routine clinical sample evaluation and transferred to the Armed Forces Research Institute of Medical Sciences (AFRIMS) in Bangkok, Thailand for confirmation by Phoenix BD and further analysis. Isolates were grown on MacConkey agar plates, trypticase soy agar plates, blood agar plates, or Mueller Hinton agar plates in preparation for analyses.

### Biochemical identification and antimicrobial susceptibility testing

Biochemical identification and antimicrobial susceptibility testing were performed with the BD Phoenix™ 50 using the NMIC/ID-4 and NMIC/ID-95 panel for Gram-negative ESKAPEE pathogens and PMIC/ID-55 for Gram-positive ESKAPEE pathogens, according to the manufacturer’s instructions (BD Diagnostics, Sparks, MD) following the CLSI guideline, 2017 [20], Additional file 1: Table S1. The colistin minimum inhibitory concentration (MIC) values were interpreted according to the European Committee on Antimicrobial Susceptibility Testing (EUCAST) guidelines, 2017 [21].

### PCR and real-time PCR assay

Genomic DNA from bacterial isolates were extracted using the QIAGEN DNeasy Blood and Tissue Kit (Germantown, MD, USA). The genomic DNA concentration and purity were measured by Nanodrop 2000c Spectrophotometer (Thermo Scientific, MA, USA). The genomic DNA at a concentration between 1 and 10 ng/µl, was used as the DNA template for PCR and real-time PCR and was stored at − 20 °C until use.

Real-time PCR assays were performed as described previously to determine the presence of carbapenemase antimicrobial resistance genes (*bla*_NDM_ and *bla*_KPC_) [22] and methicillin resistance gene (*mecA*) [23] using SensiFAST Probe No-ROX Mix (Bioline, London, UK) on CFX96 Touch Deep Well™ Real-Time PCR Detection System (Bio-Rad, CA, USA). Analysis was performed using Bio-Rad CFX Manager software version 3.1.

PCR assays were performed as described previously to determine the presence of ESBL genes [24], carbapenemase genes (*bla*_OXA-23,24,51,58_) [25], colistin resistance genes (*mcr-1* to *mcr-9*) [26, 27], vancomycin resistance genes [28], and *Enterococcus* speciation (*ddlE*) [28] using the AmpliTaq^®^ Gold DNA polymerase (Thermo Fisher Scientific, USA) on Mastercycler® nexus (Eppendorf, Inc, USA).

### DNA sequencing

The resistance genes identified from the multiplex PCR assays were sequenced to determine variant types. Genetic sequencing services were contracted through 1st BASE (Malaysia). The nucleotide sequences were analyzed with the Sequencher software version 5.3 and BLAST function on NCBI (http://www.ncbi.nlm.nih.gov/BLAST).

### Short- and long-read whole-genome sequencing

Confirmed MDR ESKAPEE isolates that were non-repeating based on AMR phenotypes, and have not been published isolates were subjected to short-read whole-genome sequencing (WGS) on an Illumina MiSeq Benchtop sequencer and selected colistin resistance isolates were subjected to long-read WGS on Pacific Biosciences RSII instrument. DNA extraction, library preparation and sequencing were performed as described previously [13]. In brief, DNA was extracted using the Ultra-Clean Microbial DNA Isolation Kit (MoBio, Inc. Carlsbad, CA). For short-read sequencing, the sequencing library was prepared and quantified using the KAPA HyperPlus Library Preparation Kit and the KAPA Library Quantification Kit-Illumina/Bio-Rad iCycler, respectively (Roche Diagnostics Corporation, Indianapolis, IN), on a CFX96 real-time cycler (Bio-Rad, Hercules, CA, USA). Sequencing of samples was performed using MiSeq Reagent Kit v3 (600 cycle; 2–300 bp) (Illumina, San Diego, CA, USA). For Long-read WGS, the library preparation and size selection were performed using PacBio Template Preparation 1.0 kit (Pacific Biosciences, Menlo Park, CA, USA) and Blue Pippin (Sage Sciences, Beverly, MA, USA), respectively. The sequencing was performed on individual cells of Pacific Biosciences RSII instrument using 240 min movies.

### Analysis of WGS data

The analysis of WGS was previously described [13]. Briefly, sequences from short-read WGS data were quality trimmed for an average quality score that was below 15 (Phred), using a sliding 5 bp window. Adaptor sequences were trimmed before performing de novo assembly with Newbler (V2.7). Minimum thresholds for contig size was set at 100 bp and a minimum coverage of 50X. de novo assembly of long-read sequencing data was performed using HGAP 2.0 in the SMRT Analysis Portal (Pacific Bioscienes). To generate circularized plasmid from long-read WGS data, overlapping contig ends were removed and short-read WGS data were mapped to circularized contigs to detect and correct errors. Geneious (Biomatters, Auckland, New Zealand) was used to perform comparative genomic analyses. Annotation of antimicrobial resistance genes was performed using ResFinder 2.0 [29]. NCBI Prokaryotic Genome Annotation Pipeline version 4.1 was used to annotate the genome.

## Results

A total of 401 confirmed Gram-negative MDR-ESKAPEE and 30 confirmed Gram-positive MDR-ESKAPEE pathogens were identified from Queen Sirikit Naval Hospital from 2017 to 2018 (Table 1). These were confirmed, non-duplicated MDR isolates that have not been published. *E. coli* (41.3%) was the most common pathogen identified, followed by *K. pneumoniae* (23.7%) and *A. baumannii* (22.5%). Antibiotic resistant phenotypes of 401 Gram-negative MDR-ESKAPEE were as follows; carbapenem resistant isolates 204/401 (51%), ESBL-only producers 182/401 (45%), ESBL producers and colistin resistant 7/401 (2%), and non-ESBL producers 8/401 (2%). Among the 204 carbapenem resistant isolates, a high percentage of *A. baumannii* (47.5%) and *K. pneumoniae* (33.4%) were identified, while the majority of *E. coli* were ESBL-only producers (82.4%). There were 6 *E. coli* and 1 *K. pneumoniae* that were both ESBL producers and colistin resistant due to the carriage of *mcr* genes*.* Eight MDR isolates (6 *E. coli* and 2 *K. pneumoniae)* that were resistant to other antibiotic classes were also identified (Table 1). Of 30 Gram-positive MDR-ESKAPEE isolates, all *E. faecium* 5/5 (100%) were VRE and 21/25 (84%) *S. aureus* were MRSA.

**Table 1:** Distribution of pathogens and antimicrobial resistance phenotypes of 431 ESKAPEE isolates collected in Chonburi, Thailand in 2017–2018

Organism	No. of ESKAPEE isolates (431)	Resistance phenotypes in Gram-negative isolates (401)				Resistance phenotypes in Gram-Positive isolates (30)	
		CRE 204 (51%)	ESBL only 182 (45%)	Colistin + ESBL 7 (2%)	MDR (non-ESBL) 8 (2%)	VRE 5 (17%)	MRSA 21 (70%)
*E. faecium*	5 (1.2%)	N/A	N/A	N/A	N/A	5 (100%)	N/A
*S. aureus*	25 (5.8%)	N/A	N/A	N/A	N/A	N/A	21 (100%)
*K. pneumoniae*	102 (23.7%)	68 (33.4%)	31 (17%)	1 (14.3%)	2 (25%)	N/A	N/A
*A. baumannii*	97 (22.5%)	97 (47.5%)	0	0	0	N/A	N/A
*P. aeruginosa*	20 (4.6%)	19 (9.3%)	1 (0.6%)	0	0	N/A	N/A
*Enterobacter* spp.	4 (0.9%)	4 (2%)	0	0	0	N/A	N/A
*E. coli*	178 (41.3%)	16 (7.8%)	150 (82.4%)	6 (85.7%)	6 (75%)	N/A	N/A
Total No. (%)	431 (100%)	204 (100%)	182 (100%)	7 (100%)	8 (100%)	5 (100%)	21 (100%)

*N/A* not available

### Antibiotic resistance phenotypes

A variety of antibiotic resistance phenotypes were identified among the collected MDR ESKAPEE pathogens. Seventy to one hundred percent of the Gram-negative isolates were resistant to β-lactam antibiotic classes (cephems, monobactams and β-lactam inhibitors), a summary of antibiotic resistant phenotypes is provided in Table 2. MDR-*E. coli* isolates were more sensitive to amikacin (96%) compared to tobramycin (59%) and gentamicin (42%), however, all *K. pneumonia* and *P. aeruginosa* were sensitive to amikacin. Six *E. coli* and 1 *K. pneumoniae* were resistant to colistin. Of these, 5 *E. coli* isolates were isolated from urine, 1 from pus, and *K. pneumoniae* was from sputum (Additional file 2: Table S2). However, the resistance to polymyxin was relatively low indicating that the resistance was not as widespread in this area. More than 94% of *A. baumannii* were resistant to five antibiotics classes (carbapenems, cephems, monobactams, β-lactam inhibitors, and 5-fluoroquinilones; Table 2). Nineteen out of 21 MRSA isolates were resistant to ciprofloxacin and 12 were resistant to tetracycline. Of note, all 21 MRSA isolates (100%) were resistant to clindamycin, 1 isolate was resistant to mupirocin and 4 isolates were resistant to Trimethoprim-Sulfamethoxazole (Table 3). All 5 VRE isolates were resistant to the 14 antibiotics tested, including the first-line treatment for VRE: ampicillin, gentamycin, and linezolid (Table 3). One VRE was resistant to linezolid but susceptible to quinupristin-dalfopristin and nitrofurantoin while another isolate was resistant to quinupristin-dalfopristin but susceptible to linezolid. No VISA or VRSA isolates were identified in this study.

**Table 2:** Resistance to tested antibiotics in Gram-negative ESKAPEE bacterial isolates

Antibiotic class	Antibiotic Name	No. resistance isolates (%) (n = 401)	*K. pneumoniae* (%) (n = 102)	*A. baumannii* (%) (n = 97)	*P. aeruginosa* (%) (n = 20)	*Enterobacter* spp. (%) (n = 4)	*E. coli* (%) (n = 178)
Carbapenem	Ertapenem	196 (49)	55 (54)	97 (100)	20 (100)	4 (100)	20 (11)
	Imipenem	178 (44)	58 (57)	95 (98)	14 (70)	0	11 (6)
	Meropenem	176 (44)	59 (58)	95 (98)	12 (60)	0	9 (5)
Cephem	Cefazolin^1^	401 (100)	102 (100)	97 (100)	20 (100)	4 (100)	178 (100)
	Cephalothin^1^	395 (98)	99 (97)	97 (100)	20 (100)	4 (100)	178 (100)
	Cefuroxime^2^	386 (96)	99 (97)	97 (100)	20 (100)	4 (100)	166 (93)
	Cefoxitin^2^	344 (86)	59 (58)	97 (100)	20 (100)	4 (100)	164 (92)
	Cefotaxime^3^	398 (99)	102 (100)	94 (97)	20 (100)	2 (50)	178 (100)
	Ceftazidime^3^	378 (94)	99 (97)	94 (97)	15 (75)	3 (75)	167 (94)
	Ceftriaxone^3^	379 (95)	97 (95)	94 (97)	20 (100)	4 (100)	164 (92)
	Cefepime^4^	373 (93)	99 (97)	94 (97)	15 (75)	3 (75)	162 (91)
Monobactam	Aztreonam	380 (95)	99 (97)	97 (100)	14 (70)	3 (75)	167 (94)
β-Lactam inhibitor	Ampicillin	397 (99)	102 (100)	97 (100)	20 (100)	4 (100)	174 (98)
	Ampicillin-Sulbactam	401 (100)	102 (100)	97 (100)	20 (100)	4 (100)	178 (100)
	Amoxicillin-Clavulanate	388 (97)	100 (98)	97 (100)	20 (100)	4 (100)	167 (94)
	Piperacillin	389 (97)	102 (100)	97 (100)	10 (50)	2 (50)	178 (100)
	Piperacillin-Tazobactam	188 (47)	71 (70)	94 (97)	6 (30)	3 (75)	14 (8)
5-Fluoroquinolone	Ciprofloxacin	348 (87)	86 (84)	94 (97)	14 (70)	3 (75)	151 (85)
	Levofloxacin	341 (85)	88 (86)	91 (94)	10 (50)	2 (50)	150 (84)
	Moxifloxacin	355 (88)	86 (84)	97 (100)	20 (100)	2 (50)	150 (84)
	Norfloxacin	349 (87)	84 (82)	94 (97)	14 (70)	4 (100)	153 (86)
Folate Antagonist	Trimethoprim	326 (82)	84 (82)	97 (100)	20 (100)	0	125 (70)
	Trimethoprim-Sulfamethoxazole	300 (75)	87 (85)	74 (76)	20 (100)	0	119 (67)
Aminoglycoside	Gentamicin	249 (62)	44 (43)	88 (91)	11 (55)	3 (75)	103 (58)
	Amikacin	94 (23)	0	85 (88)	0	2 (50)	7 (4)
	Tobramycin	228 (57)	73 (72)	69 (71)	11 (55)	2 (50)	73 (41)
Tetracycline	Tetracycline	341 (85)	94 (92)	88 (91)	20 (100)	2 (50)	137 (77)
Nitrofurantoin	Nitrofurantoin	201 (47)	61 (60)	97 (100)	20 (100)	4 (100)	7 (4)
Chloramphenicol	Chloramphenicol	196 (49)	26 (25)	97 (100)	20 (100)	0	53 (30)
Polymyxin	Colistin	7 (1.7)	1(1)	0	0	0	6 (3)

^1,2,3,4^1st, 2nd, 3rd, and 4th generation of cephalosporin

**Table 3:** Resistance to tested antibiotics in Gram-positive ESKAPEE bacteria isolates

Antibiotic class	Antibiotic name	No. resistance isolates (%) (n = 30)	*E. faecium* (%) *(*n = 5)	*S. aureus* (%) *(n* = *25)*
Aminoglycoside	Amikacin	5 (17)	5 (100)	0 (0)
	Gentamicin	14 (47)	5 (100)	14 (56)
	Tobramycin	19 (63)	5 (100)	14 (56)
β-Lactam inhibitor	Amoxicillin-Clavulanate	21 (70)	0 (0)	21 (84)
	Ampicillin	30 (100)	5 (100)	25 (100)
	Oxacillin	21 (70)	0 (0)	21 (84)
Natural penicillins	Penicillin G	25 (83)	0 (0)	25 (100)
Cephem	Cefoxitin	5 (17)	5 (100)	0 (0)
5-Fluoroquinolone	Ciprofloxacin	24 (80)	5 (100)	19 (76)
Lincosamide	Clindamycin	26 (87)	5 (100)	21 (84)
Macrolide	Erythromycin	26 (87)	5 (100)	21 (84)
Fusidane	Fusidic acid	5 (17)	5 (100)	0 (0)
Oxazolidinone	Linezolid	1 (3)	1 (20)	0 (0)
Antibacterials, Topical	Mupirocin	1 (3)	0 (0)	1 (4)
	Mupirocin High level	1 (3)	0 (0)	1 (4)
Nitrofurantoin	Nitrofurantoin	4 (13)	4 (80)	0 (0)
Streptogramin	Quinupristin-dalfopristin	1 (3)	1 (20)	0 (0)
Ansamycin	Rifampin	2 (7)	0 (0)	2 (8)
Tetracycline	Tetracycline	17 (57)	5 (100)	12 (48)
Folate Antagonist	Trimethoprim	10 (33)	5 (100)	5 (20)
	Trimethoprim-Sulfamethoxazole	9 (30)	5 (100)	4 (16)
Glycopeptide	Vancomycin	5 (17)	5 (100)	0 (0)
	Teicoplanin	5 (17)	5 (100)	0 (0)

### Antibiotic resistance genotypes

A wide variety of antibiotic resistance genes were identified from the isolates collected in this study, including all five major carbapenemase resistance genes (*bla*_KPC_, *bla*_NDM_, *bla*_OXA-48-like_, *bla*_IMP_ and *bla*_VIM_) and two colistin resistance genes (*mcr-1* and *mcr-3)*. The majority of *A. baumannii* isolates harbored *bla*_OXA-23_ (94/97, 97%), which represents 23% of all Gram-negative isolates collected. *K. pneumoniae*, *E. coli, P. aeruginosa* and *Enterobacter* spp. isolates were shown to harbor *bla*_NDM_ (69/401, 17%) and *bla*_OXA-48-like_ (51/401, 13%) carbapenemase genes, with *K. pneumoniae* carrying the highest percentage (Table 4). *K. pneumoniae* and *P. aeruginosa* isolates were shown to harbor *bla*_IMP_ (1/201, 0.5%) and *bla*_VIM_ (2/20, 10%), respectively. ESBL-producing isolates harbored a variety of ESBL genes with *bla*_CTX-M_ group 1 (182/401, 45%) being the most common in *K. pneumoniae, A. baumannii*, and *E. coli* (Table 4).

**Table 4:** Antibiotic resistance genes in ESKAPEE bacteria isolates

Resistance phenotypes	AMR genes	*E. faecium (*n = 5)	*S. aureus* (n = 25)	*K. pneumoniae* (n = 102)	*A. baumannii* (n = 97)	*P. aeruginosa* (n = 20)	*Enterobacter* spp. (n = 4)	*E. coli* (n = 178)	Total (GN^a^ = 401, GP^b^ = 30)
Carbapenemase genes	NDM	N/A	N/A	56 (55%)	4 (4%)	1 (5%)	1 (25%)	7 (4%)	69 (17%)
	KPC	N/A	N/A	0	0	0	0	1 (0.5%)	1 (0.25%)
	OXA-48 like	N/A	N/A	48 (47%)	0	0	0	2 (1%)	50 (13%)
	IMP	N/A	N/A	1 (1%)	0	0	0	0	1 (0.25%)
	VIM	N/A	N/A	0	0	2 (10%)	0	0	2 (0.5%)
	OXA-23	N/A	N/A	0	93 (97%)	0	0	0	93 (23%)
	OXA-24	N/A	N/A	0	0	0	0	0	0
β-lactamase genes	TEM	N/A	N/A	84 (82%)	77 (79%)	0	3 (75%)	63 (35%)	227 (57%)
	SHV	N/A	N/A	31 (30%)	0	0	0	0	31 (8%)
	OXA	N/A	N/A	18 (18%)	0	0	3 (75%)	42 (24%)	63 (16%)
	CTX-M group 1	N/A	N/A	91 (89%)	2	0	3 (75%)	86 (48%)	182 (45%)
	CTX-M group 9	N/A	N/A	4 (4%)	0	0	0	68 (38%)	72 (18%)
	CTX-M group 8/25	N/A	N/A	1 (1%)	0	0	0	0	1 (0.25%)
	DHA	N/A	N/A	4 (4%)	0	0	0	4 (2%)	8 (2%)
	CMY	N/A	N/A	14 (14%)	0	0	0	13 (7%)	27 (7%)
	PER	N/A	N/A	0	3 (3%)	1 (5%)	0	0	4 (1%)
	VEB	N/A	N/A	0	2 (2%)	5 (25%)	0	1 (0.5%)	8 (2%)
Colistin resistance genes	*mcr-1*	N/A	N/A	1 (1%)	0	0	0	3 (1.6%)	4 (1%)
	*mcr-3*	N/A	N/A	0	0	0	0	1 (0.5%)	1 (0.25%)
	*mcr-1*, *mcr-3*	N/A	N/A	0	0	0	0	2 (1%)	2 (0.5%)
Methicillin resistance gene	*mecA*	N/A	21 (84%)	N/A	N/A	N/A	N/A	N/A	21 (70%)
Vancomycin resistance gene	*vanA*	5 (100%)	N/A	N/A	N/A	N/A	N/A	N/A	5 (17%)

^a^GN: Gram-negative bacteria

^b^GP: Gram-Positive bacteria

*N/A* not applicable, OXA-48 like: OXA-181, OXA-232 and OXA-247; CTX-M group 1: CTX-M-15 and CTX-M-55; CTX-M group 9: CTX-M-14, CTX-M-24, and CTX-M-27; CTX-M group 8/25: CTX-M-63

All 431 short-read WGS data and 2 long-read WGS data of plasmid in *E. coli* with *mcr* genes were submitted to NCBI database and is available as BioProject: PRJNA814829. The MLST data of WGS, BioSample accession number, and other information about bacterial isolates are reported in Additional file 3: Table S3. The sample ID of 431 isolates with AMR genes detected by WGS are reported in Additional file 4: Table S4. The combined WGS data of colistin-resistant *E. coli* isolates will be further analysed via de novo assembly. ESBL genes were common among the collected *E. coli* isolates and WGS analysis further identified *bla*_NDM-4_ in 1 isolate, *bla*_NDM-5_ in 6 isolates, and *bla*_KPC-2_ in 1 isolate. Notably, of the 178 *E. coli* isolates, 151 isolates carried at least one of the five common CTX-M genes: *bla*_CTX-M_ group 1 (*bla*_CTX-M-15_, *bla*_CTX-M-55_) or *bla*_CTX-M_ group 9 (*bla*_CTX-M-14_, *bla*_CTX-M-24_, and *bla*_CTX-M-27_). Three isolates carried both *bla*_CTX-M_ group 1 and group 9 genes. Forty-two isolates carried *bla*_OXA-1_. Thirteen isolates carried AmpC β-lactamase *bla*_CMY_. Four isolates carried AmpC β-lactamase *bla*_DHA_ (Table 4). Of note, the 16S methyltransferase *rmtB*, which confers resistance to all clinically relevant aminoglycosides, was identified in two isolates. Six *E. coli* isolates carried the colistin resistant genes: *mcr-1*, *mcr-3*, or both *mcr-1* and *mcr-3* (Table 4). The six isolates also harbored *bla*_CTX-M-55_.

WGS data analysis of 102 K*. pneumoniae* isolates identified 80 antibiotic resistance genes, including the carbapenemases *bla*_NDM-1_, *bla*_NDM-5_, variants of *bla*_OXA-48-like_, and 1 isolate carried *bla*_IMP-15_. The majority of *K. pneumoniae* isolates carried a variant of the CTX-M genes, with 91 out of 102 carrying *bla*_CTX-M-15_. One *K. pneumoniae* isolate carried *bla*_CTX-M-15_ and *bla*_CTX-M_ group 8/25, which was identified to be *bla*_CTX-M-63_ by WGS. Of particular concern was the high prevalence of the carbapenemase genes identified in *K. pneumoniae* (48/102), compared to other Gram-negative species, with both *bla*_NDM-1_ and one of the three *bla*_OXA-48-like_ variants: *bla*_OXA-181_, *bla*_OXA-232_, and *bla*_OXA-247_ identified. A number of isolates carried multiple aminoglycoside modifying enzymes (AMEs) that confer resistance to gentamicin and tobramycin but no 16S methyltransferase were identified. One *K. pneumoniae* carried the colistin resistant gene, *mcr-1* and *bla*_CTX-M-55_ (Table 4).

Based on the antibiotic resistance profile, 93 out of 97 *A. baumannii* strains carried a combination of carbapenem-resistant genes with 93 isolates harboring the most prevalent Class D carbapenemase, *bla*_OXA-23_, which has been reported to be associated with carbapenem resistance *A. baumannii* worldwide [30]. Two isolates also carried an additional *bla*_OXA-58_, a plasmid-borne resistance gene which has been shown to contribute significantly to carbapenem resistance in *A. baumannii* [31]. Four isolates carried the potent carbapenemase resistance gene, *bla*_NDM-1_ (Table 4). Notably, 71 isolates also carried *armA* (aminoglycoside resistance methylase), which confers resistance to all clinically relevant aminoglycosides.

WGS analysis of *P. aeruginosa* identified 59 antibiotic resistance genes. Two carbapenemase genes were also detected in 3 isolates, 1 isolate carried *bla*_NDM_ and 2 isolates carried *bla*_VIM_ genes (Table 4). Two isolates also carried a gene encoding for the 16S methyltransferase, *rmtE,* which confers resistance to aminoglycosides. ESBL producing strains were also identified, with 5 isolates carrying *bla*_VEB_ (Table 4).

The 4 *Enterobacter* spp. isolates were identified to be *E. aerogenes* (current name: *Klebsiella aerogenes* [32]*,* 1 isolate), *E. cloacae* (1 isolate), and *E. hormaechei* (2 isolates). The *E. aerogenes* isolate carried *bla*_NDM-1_. Only *bla*_CTX-M-15_ was identified in the remaining *Enterobacter* spp. (Table 4). Although the *Enterobacter* spp. in this study do not carry a large number resistance genes, they harbor genes that confer resistance to clinically significant antibiotic classes.

*E. faecium* isolates identified by *ddlE* gene were confirmed as *E. faecium* by WGS, which revealed 18 different loci of antibiotic resistance genes including the six genes that comprise the vancomycin resistance operon (*vanS*, *vanR*, *vanH*, *vanA*, *vanB* and *vanX*) [33]. *S. aureus* isolates (21/25, 84%) were positive for the *mecA* gene indicating a significant presence of MRSA in studied isolates (Table 4). From WGS analysis, *qacA* and *qacB* genes, conferring tolerance to quaternary ammonium disinfectants, were identified in 4 and 5 MRSA isolates, respectively. Moreover, 1 MRSA isolate also carried the *mupA* gene, which is involved in high-level resistance to mupirocin, (Table 3).

## Discussion

### Carbapenem-resistant *Enterobacteriaceae* (CRE)

CRE are a dangerous global health issue due to resistance against antibiotic classes used to treat severe infections and the potential for a widespread transmission of carbapenem resistance genes via mobile genetic elements [34]. The most important carbapenemases among *Enterobacteriaceae* clinical isolates are KPC and NDM. While KPC-producing organisms are rarely reported in Asian countries [35, 36], NDM-1 carbapenemase producing *Enterobacteriaceae* strains have been detected worldwide [37].

In this study, > 50% of *K. pneumoniae* isolates were resistant to carbapenems, while the majority of *E. coli* isolates (89–95%) were susceptible (Table 2). In recent years, many *K. pneumoniae* have acquired a variety of β-lactamase enzymes, which hydrolyze β-lactam antibiotics such as penicillins, cephalosporins, and carbapenems. In *K. pneumoniae,* the emergence of NDM-1, encoded by *bla*_NDM-1_, has resulted in an increase of carbapenem-resistant isolates that carry this gene. Strains that carry *bla*_NDM-1_ have a resistance profile that poses a critical threat to effective clinical treatment and often requires additional antibiotic treatment with aminoglycosides and fluoroquinolones [38].

While most *E. coli* are commensal and constitute approximately 0.1% of the normal intestinal flora, some strains are inherently pathogenic. Within the last decade, *E. coli* has shown a marked resistance expansion to multiple antibiotics, reaching or exceeding the non-susceptibility profile of *K. pneumoniae* [39, 40]. Through horizontal transfer of resistance genes, *E. coli* can acquire multiple resistance traits and has the potential for an even greater clinical impact than that of carbapenem-resistant *K. pneumoniae* due to being responsible for a wide range of infections in humans [39]. A lower rate of MDR *E. coli* demonstrated carbapenem resistance in this study compared to other Gram-negative species. Carbapenems (ertapenem, imipenem, meropenem) were still largely effective against collected *E. coli* isolates, including some β-lactam antibiotics (cephalothin, piperacillin/tazobactam; Table 2). However, the prevalence of carbapenem resistance among MDR *E. coli* in this study (ertapenem 11%, imipenem 6%, meropenem 5%) was higher than the antimicrobial resistance rate of *E. coli* reported by NARST during 2000–2020, (ertapenem 2.4%, imipenem 2%, meropenem 2.1%) and the increase in carbapenem resistance should continue to be monitored [9].

### ESBL-producing *Enterobacteriaceae*

Extended-spectrum beta-lactamase-producing *Enterobacteriaceae* (ESBL-PE) are resistant antibiotics commonly used as first-line treatment options and represent a major problem in the management of hospital acquired infections. The prevalence of ESBL-PE varies widely between geographic areas. Low prevalence rates have been reported in Europe, USA and North America [41], while high rates have been observed in South America, Asia [33] and some African countries [42]. *E. coli* and *K. pneumoniae* were the most common ESBL-PE isolates identified in this study and were mostly isolated from urine samples (see Additional file 1: Table S1), similar to previous findings from India [43]. Moreover, hospitalization has been identified as a high-risk factor for ESBL-PE infection, because ESBL-encoding genes are carried in plasmids that can be easily disseminated among the different bacterial species that colonize hospitalized patients [44, 45].

A significant percentage of *K. pneumoniae* and *E. coli* isolates (> 90%) from this study were phenotypically resistant to β-lactams and piperacillin-tazobactam by *E. coli* (Table 2). CTX-M group-1 (*bla*_CTX-M-15_ and *bla*_CTX-M-55_) was the most common gene family detected in samples collected in this study from the eastern region of Thailand. The CTX-M family, particularly CTX-M-15, has emerged worldwide, and is now the most common ESBL type in hospitals and in the community [46]. This data is consistent with the reported prevalence of ESBL genes at Siriraj Hospital and Thammasat Hospital that collected from the central region of Thailand [47]. In addition to CTX-M group-1, *E. coli* isolates also harbored CTX-M group-9 (*bla*_CTX-M-14_) which is another common and widely spread CTX-M group worldwide [48].

### Colistin resistance among ESKAPEE pathogens

In human clinical samples, *mcr-1* has been most frequently identified in *E. coli*, but reported prevalence is increasing in other bacterial species, such as *K. pneumoniae* [49]. In Thailand, the detection of *mcr-1* has been reported in both *E. coli* isolated from environmental and human clinical samples [50–52]. We previously reported the identification of *mcr-1* in 2 *K. pneumoniae* with one also carrying 2 carbapenemase genes (*bla*_NDM-1_ and *bla*_OXA-232_), a potent resistance combination that results in difficult to treat infections in a clinical setting [13]. This study reports the co-occurrence of *mcr-1* and *mcr-3* in *E. coli* clinical isolates. However, the co-occurrence of *mcr-1* and *mcr-3* genes in *E.coli* strains did not affect the level of resistance to colistin (MIC = 4 µg/mL) when compared to isolates that carried only *mcr-1* or *mcr-3*. These isolates also carry an ESBL gene resistance background, *bla*_CTX-M-55_, which is a concerning combination for selecting effective treatments. Additional analysis to further characterize and investigate the co-occurrence is underway. These findings indicate that the circulation of *mcr-1* and *mcr-3* may be a more common occurrence in hospital settings than previously recognized.

### Multidrug-resistant *A. baumannii* and *P. aeruginosa*

The rate of carbapenem-resistant *A. baumannii* and *P. aeruginosa* have been previously reported to be high in Asian countries [53]. Surveillance conducted in the Asia–Pacific region reported that 73% of *A. baumannii* and 29.8% of *P. aeruginosa* were resistant to 2 out of 3 carbapenems tested compared to other *Enterobacteriaceae* species that were 97.2% (or only 3% resistant) susceptible to all carbapenems tested [54]. In this study, a significantly higher percentages of *A. baumannii* and *P. aeruginosa*, 98% and 60%, respectively, were resistant to 2 out of 3 carbapenems tested. *A. baumannii* and *P. aeruginosa* isolates in this study displayed a consistent carbapenem resistance trend, harboring a variety of carbapenemase resistance genes including *bla*_NDM_ and *bla*_VIM_ (Table 4). Both microorganisms carry intrinsic antibiotic resistance mechanisms with the potential to acquire new antibiotic resistance genes carried on mobile genetic elements.

A high percentage of *A. baumannii* and *P. aeruginosa* β-lactamase producers has been noted in this study but there was a relatively low prevalence of ESBL genes demonstrating that ESBL genes are not commonly harbored by *A. baumannii* and *P. aeruginosa* in this region. Although the common mechanisms of resistance is the production of beta-lactamases and aminoglycoside-modifying enzymes, diminished expression of outer membrane proteins, mutations in topoisomerases, and up-regulation of efflux pumps also play an important role in antibiotic resistance [55]. The accumulation of multiple mechanisms of resistance has led to the development of resistance to multiple antibiotics and “pan-resistant” microorganisms [55]. *A. baumannii* and *P. aeruginosa* also remain problematic because of their high intrinsic resistance to a wide variety of antimicrobial agents including cephem and β-lactam inhibitor [56], however, the presence of *bla*_NDM-1_ in these isolates is concerning.

### Multidrug-resistant* Enterobacter* species

*Enterobacter* spp. have become an increasing concern as serious hospital acquired infections have been shown to be caused by *Enterobacter* spp. with plasmid-borne ESBLs and carbapenemases [57]. Besides colistin and tigecycline, few antimicrobials are effective against resistant *Enterobacter* spp. and many of the other ESKAPEE pathogens [58]. Only a small number of MDR *Enterobacter* spp. isolates were identified in this study, but all were resistant to major classes of antibiotics (carbapenem, cephem, β-lactam inhibitor, 5’fluoroquinolone and aminoglycoside) and harbored key resistance genes (*bla*_NDM_, *bla*_CTX-M_ group 1 and *bla*_OXA_) that are of concern for drug-resistance among *Enterobacter* spp.

### Multidrug-resistant Gram-positive ESKAPEE pathogens

VRE emerged in North America during the late 1980s, with 61% of *E. faecium* isolates estimated to be vancomycin resistant by 2002 [39]. All 5 VRE isolates from this study were positive for *vanA*, which is consistent with other reports for clinical isolates in Asia [33]. In Thailand, a total of 10,470 clinical enterococcal isolates from patients from Rajvithi Hospital collected between 1999 and 2009 were evaluated, and 199 (99%) *E. faecium* and 2 (0.1%) *E. faecalis* were VRE with both *vanA* and *vanB* detected [59]. Although the number of *Enterococcus* isolates in this study was low, they were highly resistant to primary antibiotics used in the treatment of VRE with a complete resistance to ampicillin and gentamicin and only 1 isolate resistant to linezolid (Table 2). Most VRE isolates were also resistant to 2nd line antibiotics, with 4 isolates resistance to nitrofurantoin and 1 isolate resistance to quinupristin-dalfopristin [60]. VRE in this study differed in their sensitivity to various antibiotics tested indicating that treatment for VRE infection must be individualized based on the resistance and susceptibility patterns of the isolates.

MRSA is typically resistant to all β-lactams, but resistance to other antibiotic classes varies [61]. This pattern was present for *S. aureus* in this study with 100% susceptibility to glycopeptide antibiotics (Table 3). A high percentage of *S. aureus* with *mecA* (84%) in this study is alarming as the treatment may require glycopeptide antibiotics, and thus contribute to the rise of the existing problem of VISA and VRSA. Asian countries have shown very high rates (> 50%) of MRSA, which is a major cause of hospital-acquired infections such as pneumonia, surgical site infections, and bloodstream infections [62]. Multiple studies in Thailand have reported a variable percentage of MRSA prevalence ranging from 46%-61% with hospital-associated MRSA as the most common source of MRSA [2, 63, 64]. The presence of *qacA/B* and *mupA*, conferring tolerance to quaternary ammonium disinfectants and resistance to high level of mupirocin, respectively, is also of concern as one of the strategies to prevent MRSA transmission is the use of chlorhexidine gluconate and mupirocin-based prevention [23]. Added surveillance efforts to monitor these genes may be beneficial to the control and management of MRSA.

## Conclusion

This study reports the collection of 431 MDR ESKAPEE pathogens from Chonburi province, Thailand, collected from 2017 to 2018. The most prevalent carbapenemase gene detected was *bla*_OXA-23_, followed by *bla*_NDM_ and *bla*_OXA-48-like_. The most prevalent ESBL gene was *bla*_CTX-M group 1_, followed by *bla*_CTX-M group 9_, with both most commonly carried by *K. pneumoniae* and *E. coli* isolates*.* Colistin resistance genes were detected, including a co-occurrence of *mcr-1* and *mcr-3* in 2 *E. coli* isolates. Finally, *vanA* and *mecA* genes were prevalent in VRE and MRSA isolates, respectively. The abundance and variety of antibiotic resistance genes and MDR microorganisms identified in this study is unsurprising but still alarming, especially due to the noted increasing trend of MDR bacterial isolates in Thailand [8]. The findings from this study indicate an ongoing struggle to control the spread of AMR pathogens in Thailand, which presents ever increasing challenges to clinicians, scientists, infected individuals, and the public health system. Continuous surveillance is an essential tool for understanding of the magnitude and future trends of AMR infections, as this information can be incorporated and developed into future strategic plans for antibiotic usage and infection control procedures based on multifaceted collaborations among all relevant regional and global stakeholders.

## Supplementary Information

**Additional file 1: Table S1. **The antimicrobial agents and concentrations in NMIC/ID-4 panel, NMIC/ID-95 panel and PMIC/ID-55 panel.

**Additional file 2: Table S2. **Sources of specimen of 431 ESKAPEE bacteria isolates.

**Additional file 3: Table S3. **Bacterial isolates and MLST results.

**Additional file 4: Table S4 A-G. **Whole genome sequencing data of 431 MDR ESKAPEE isolates.

## Data Availability

The datasets used and/or analyzed during the current study are available in the NCBI under BioProject: PRJNA814829 repository.

## Abbreviations

AFRIMS: Armed Forces Research Institute of Medical Sciences
AMR: Antimicrobial resistance
BLAST: Basic Local Alignment Search Tool
CLSI: Clinical and Laboratory Standards Institute
CRE: Carbapenem-resistant Enterobacteriaceae
ESBLs: Extended-spectrum beta-lactamases
ESKAPEE: *Enterococcus faecium*, *Staphylococcus aureus*, *Klebsiella pneumoniae*, *Acinetobacter baumannii*, *Pseudomonas aeruginosa*, *Enterobacter* species and *Escherichia coli*
EUCAST: European Committee on Antimicrobial Susceptibility Testing
MDR: Multi-drug resistant
MIC: Minimum inhibitory concentration
MRSA: Methicillin-resistant *S. aureus*
MRSCN: Methicillin-resistant *Staphylococcus* coagulase negative
MRSN: Multidrug-Resistant Organism Repository and Surveillance Network
NARST: National Antimicrobial Resistance Surveillance Center, Thailand
NCBI: National Center for Biotechnology Information
PCR: Polymerase chain reaction
QSH: Queen Sirikit Naval Hospital
SNP: Single-nucleotide polymorphism
VRE: Vancomycin-resistant Enterococci
VISA: Vancomycin-intermediate *S. aureus*
VRSA: Vancomycin-resistant *S. aureus*
WGS: Whole genome sequencing
