# Supplementary material for: Molecular characterization of multidrug-resistant ESKAPEE pathogens from clinical samples in Chonburi, Thailand (2017–2018)
Source: BMC Infect Dis. 2022 Aug 17;22:695. doi: 10.1186/s12879-022-07678-8 (PMC9386987; doi:10.1186/s12879-022-07678-8)
Supplement: Supplementary file 1 — Additional file 1: Table S1. The antimicrobial agents and concentrations in NMIC/ID-4 panel, NMIC/ID-95 panel and PMIC/ID-55 panel. [file 12879_2022_7678_MOESM1_ESM.pdf]

Table S1. The antimicrobial agents and concentrations in NMIC/ID-4 panel, NMIC/ID-95 panel and PMIC/ID-55 panel.

NMIC/ID-4* REF **448505**

Panel Contents		Quality Control Organisms and Expected Results				
**Antimicrobic**	**Code**	**Conc. Range (µg/mL)**	***E.coli* ATCC 25922**	***P. aeruginosa* ATCC 27853**	***E.coli* ATCC 35218**	***K. pneumoniae* ATCC 700603**
Amikacin	AN	8 – 32	≤0.5 – 4	1 – 4	–	–
Amoxicillin/Clavulanate	AMC	4/2 – 16/8	2/1 – 8/4	–	4/2 – 16/8	–
Ampicillin	AM	4 – 16	2 – 8	–		–
Ampicillin/Sulbactam	SAM	4/2 – 16/8	2/1 – 8/4	–	8/4 – 32/16	–
Aztreonam	ATM	2 – 16	≤0.5	2 – 8	–	–
Cefazolin	CZ	4 – 16	1 – 4	–	–	–
Cefepime	FEP	2 – 16	≤0.5	0.5 – 4	–	–
Cefotaxime	CTX	1 – 32	≤0.5	8 – 32	–	–
Ceftazidime	CAZ	1 – 16	≤0.5	1 – 4	–	–
Chloramphenicol	C	4 – 16	2 – 8	–	–	–
Ciprofloxacin	CIP	0.5 – 2	≤0.125	0.25 – 1	–	–
Colistin	CL	0.5 – 2	≤0.5 – 2	≤0.5 – 4	–	–
ESBL	ESBL	–	NEG	–	–	POS
Gentamicin	GM	2 – 8	≤0.5 – 1	≤0.5 – 2	–	–
Imipenem	IPM	1 – 8	≤0.25	1 – 4	–	–
Levofloxacin	LVX	1 – 8	≤0.25	0.5 – 4	–	–
Meropenem	MEM	1 – 8	≤0.25	≤0.25 – 1	–	–
Moxifloxacin	MXF	1 – 4	≤0.125	1 – 8	–	–
Piperacillin	PIP	4 – 64	1 – 4	1 – 8	–	–
Piperacillin/Tazobactam	TZP	4/4 – 64/4	1/4 – 4/4	1/4 – 8/4	≤0.5/4 – 2/4	
Tetracycline	TE	2 – 8	≤0.5 – 2	8 – >16	–	–
Trimethoprim/Sulfamethoxazole	SXT	0.5/9.5 – 2/38	≤0.5/9.5	8/152 – >16/304	–	–
Cefotaxamine/Clavulanate (ESBL)	CCX	<9	N/A	–	–	N/A
Ceftazidime/Clavulanate (ESBL)	CCZ	<9	N/A	–	–	N/A
Cefpodoxime-proxetil (ESBL)	CPD	<9	N/A	–	–	N/A
Ceftazidime (ESBL)	CAZ	<9	N/A	–	–	N/A
Ceftriaxone/Clavulanate (ESBL)	CCR	<9	N/A	–	–	N/A

*Refer to the BD Phoenix System User's Manual.

NMIC/ID-95* **REF 448783**

Panel Contents		Quality Control Organisms and Expected Results				
**Antimicrobic**	**Code**	**Conc. Range (µg/mL)**	***E. coli* ATCC 25922**	***P. aeruginosa* ATCC 27853**	***E. coli* ATCC 35218**	***K. pneumoniae* ATCC 700603**
Amoxicillin/Clavulanate	AMC	4/2 – 16/8	2/1 – 8/4	–	4/2 – 16/8	–
Ampicillin	AM	4 – 16	2 – 8	–	–	–
Aztreonam	ATM	2 – 16	<0.5	2 – 8	–	–
Cefepime	FEP	1 – 16	<0.5	0.5 – 4	–	–
Cefoxitin	FOX	4 – 16	2 – 8	–	–	–
Ceftazidime	CAZ	2 – 16	≤0.5	1 – 4	–	–
Ceftriaxone	CRO	1 – 16	≤0.5	8 – 64	–	–
Cefuroxime	CXM	4 – 16	2 – 8	–	–	–
Cephalothin	CF	4 – 16	4 – 16	–	–	–
Chloramphenicol	C	4 – 16	2 – 8	–	–	–
Ciprofloxacin	CIP	0.5 – 2	≤0.125	0.25 – 1	–	–
Colistin	CL	1 – 4	≤0.5 – 2	≤0.5 – 4	–	–
Ertapenem	ETP	0.25 – 1	≤0.25	–	–	–
Gentamicin	GM	2 – 8	≤0.5 – 1	≤0.5 – 2	–	–
Imipenem	IPM	1 – 8	≤0.25	1 – 4	–	–
Meropenem	MEM	1 – 8	≤0.25	≤0.25 – 1	–	–
Nitrofurantoin	FM	16 – 64	≤8 – 16	–	–	–
Norfloxacin	NOR	2 – 8	≤0.25	1 – 4	–	–
Piperacillin/Tazobactam	TZP	4/4 – 64/4	1/4 – 4/4	1/4 – 8/4	≤0.5/4 – 2/4	–
Tobramycin	NN	2 – 8	0.25 – 1	0.25 – 1	–	–
Trimethoprim	TMP	1 – 8	≤0.5 – 2	>16	–	–
Trimethoprim/Sulfamethoxazole	SXT	0.5/9.5 – 2/38	≤0.5/9.5	8/152 – >16/304	–	–
ESBL	ESBL	–	NEG	–	–	POS
Cefotaxime/Clavulanate (ESBL)	CCX	<9	N/A	–	–	N/A
Ceftazidime/Clavulanate (ESBL)	CCZ	<9	N/A	–	–	N/A
Cefpodoxime-proxetil (ESBL)	CPD	<9	N/A	–	–	N/A
Ceftazidime (ESBL)	CAZ	<9	N/A	–	–	N/A
Ceftriaxone/Clavulanate (ESBL)	CCR	<9	N/A	–	–	N/A

*Refer to the BD Phoenix System User's Manual.

Panel Contents		Quality Control Organisms and Expected Results				
**Antimicrobic**	**Code**	**Conc. Range (µg/mL)**	***S. aureus* ATCC 29213**	***E. faecalis* ATCC 29212**	***E. faecalis* ATCC 51299**	***S. aureus* ATCC 25923**
Amikacin	AN	8 – 32	1 – 8	–	–	–
Amoxicillin/Clavulanate	AMC	1/0.5 – 4/2	≤0.25/0.125 – 0.5/0.25	≤0.25/0.125 – 1/0.5	–	–
Ampicillin	AM	2 – 8	0.5 – >1	0.5 – 2	–	–
Beta-Lactamase (Nitrocefin-based)	NCF	<10	POS	–	–	NEG
Cefoxitin	FOX	2 – 8	≤1 – 4	–	–	–
Ciprofloxacin	CIP	0.25 – 4	≤0.125 – 0.5	0.5 – 4	–	–
Clindamycin	CC	0.5 – 2	≤0.125 – 0.25	4 – >8	–	–
Erythromycin	E	0.25 – 4	0.25 – 1	–	–	–
Fusidic Acid	FA	1 – 8	≤0.5 – 1	1 – 4	–	–
Gentamicin	GM	1 – 8	≤0.5 – 1	–	–	–
Gentamicin-Synergy	GMS	500	–	≤500	>500	–
Linezolid	LZD	0.5 – 4	1 – 4	1 – 4	–	–
Mupirocin	MUP	1 – 4	≤0.5	>8	–	–
Mupirocin-High Level	MUH	256	≤256	≤256	–	–
Nitrofurantoin	FM	16 – 64	≤16 – 32	≤16	–	–
Oxacillin	OX	0.25 – 2	0.125 – 0.5	>4	–	–
Penicillin	P	0.0625 – 0.25	0.25 – >1	1 – 4	–	–
Quinupristin/Dalfopristin	SYN	0.5 – 2	≤0.5 – 1	2 – >4	–	–
Rifampin	RA	0.5 – 2	≤0.25	–	–	–
Teicoplanin	TEC	1 – 16	≤0.5 – 1	≤0.5	–	–
Tetracycline	TE	0.5 – 8	≤0.5 – 1	8 – >16	–	–
Tobramycin	NN	2 – 8	≤1	–	–	–
Trimethoprim	TMP	0.5 – 2	1 – 4	≤0.5 – 1	–	–
Trimethoprim/Sulfamethoxazole	SXT	1/19 – 4/76	≤0.5/9.5	≤0.5/9.5	–	–
Vancomycin	VA	1 – 16	≤0.5 – 2	1 – 4	–	–

*Refer to the BD Phoenix System User's Manual.
