# Supplementary material for: Molecular characterization of multidrug-resistant ESKAPEE pathogens from clinical samples in Chonburi, Thailand (2017–2018)
Source: BMC Infect Dis. 2022 Aug 17;22:695. doi: 10.1186/s12879-022-07678-8 (PMC9386987; doi:10.1186/s12879-022-07678-8)
Supplement: Supplementary file 2 — Additional file 2: Table S2. Sources of specimen of 431 ESKAPEE bacteria isolates. [file 12879_2022_7678_MOESM2_ESM.docx]

**Table S2.** Sources of specimen of 431 ESKAPEE bacteria isolates.

| **Specimen type** | **Organism** | | | | | | | |
| --- | --- | --- | --- | --- | --- | --- | --- | --- |
|  | ***E. faecium* n (%)** | ***S. aureus* n (%)** | ***K. pneumoniae n (%)*** | ***A. baumannii n (%)*** | ***P. aeruginosa n (%)*** | ***Enterobacter spp. n (%)*** | ***E. coli***  ***n (%)*** | **Total (%)** |
| Urine | 4 (2.4) | 1 (0.6) | 36 (21.4) | 15 (8.9) | 6 (3.6) | 2 (1.2) | 104 (61.9) | **168** |
| Sputum | 0 (0) | 10 (8.1) | 37 (29.8) | 49 (39.5) | 12 (9.7) | 0 | 16 (12.9) | **124** |
| Pus | 0 (0) | 8 (15.1) | 5 (9.4) | 21 (39.6) | 2 (3.8) | 1 (1.9) | 16 (30.2) | **53** |
| Blood | 0 (0) | 2 (4.4) | 7 (15.6) | 7 (15.6) | 0 (0) | 0 (0) | 29 (64.4) | **45** |
| Rectal swab | 0 (0) | 0 (0) | 13 (92.9) | 1 (7.1) | 0 (0) | 0 (0) | 0 (0) | **14** |
| Abdomen fluid | 1 (10) | 2 (20) | 1 (10) | 0 (0) | 0 (0) | 1 (10) | 5 (50) | **10** |
| Wound | 0 (0) | 0 (0) | 0 (0) | 2 (50) | 0 (0) | 0 (0) | 2 (50) | **4** |
| Stool | 0 (0) | 0 (0) | 2 (66.7) | 0 (0) | 0 (0) | 0 (0) | 1 (33.3) | **3** |
| Urinary catheter | 0 (0) | 0 (0) | 1 (33.3) | 0 (0) | 0 (0) | 0 (0) | 2 (66.7) | **3** |
| Tissue | 0 (0) | 0 (0) | 0 (0) | 1 (50) | 0 (0) | 0 (0) | 1 (50) | **2** |
| Central line | 0 (0) | 1 (50) | 0 (0) | 1 (50) | 0 (0) | 0 (0) | 0 (0) | **2** |
| Hemoculture | 0 (0) | 0 (0) | 0 (0) | 0 (0) | 0 (0) | 0 (0) | 1 (100%) | **1** |
| Bile | 0 (0) | 0 (0) | 0 (0) | 0 (0) | 0 (0) | 0 (0) | 1 (100%) | **1** |
| Content | 0 (0) | 1 (100%) | 0 (0) | 0 (0) | 0 (0) | 0 (0) | 0 (0) | **1** |
| **Total (%)** | **5 (1.2)** | **25 (5.8)** | **102 (23.7)** | **97 (22.5)** | **20 (4.6)** | **4 (0.9)** | **178 (41.3)** | **431 (100)** |
